# Subsequent Development of Desmoid Tumor after a Resected Gastrointestinal Stromal Tumor

**DOI:** 10.1155/2018/1082956

**Published:** 2018-05-02

**Authors:** Areen Abdulelah Murshid, Hatim Q. Al-Maghraby

**Affiliations:** ^1^Department of Pathology, King Abdulaziz University Hospital, Jeddah, Saudi Arabia; ^2^Department of Pathology and Laboratory Medicine, King Saud Bin Abdulaziz University for Health Sciences, King Abdulaziz Medical City, Jeddah, Saudi Arabia

## Abstract

Desmoid tumors (deep fibromatosis) of the mesentery are rare mesenchymal tumors. They are often misdiagnosed, especially with a previous history of resection for gastrointestinal stromal tumor (GIST). Immunohistochemistry can help differentiate between these two tumors. In this article, we present a case we had encountered: a Desmoid tumor developing in a patient with a history of GIST 3 years ago. It is the first case of GIST with subsequent development of Desmoid tumor to be reported in Saudi Arabia. We discuss the two entities of Desmoid tumor and GIST by comparing their definitions, clinical presentations, histological features, immunohistochemistry stains, molecular pathogenesis, prognosis, and treatment. We also discuss the relationship between GIST and the subsequent development of Desmoid tumors and compare our case with case reports in literature.

## 1. Introduction

Desmoid tumors of the mesentery are the most common primary mesenteric tumors displaying spindle cell morphology although they are very rare. They can occur in any age and can be multiple or solitary. The most encountered risk factor for developing a mesenteric Desmoid tumor is previous abdominal surgery. GIST can also develop in the mesentery and mimic clinical and radiological features of Desmoid tumors. Histologically, both tumors may look alike. We describe here a case of GIST with subsequent development of Desmoid tumor.

## 2. Case Report

**Figure 1:**
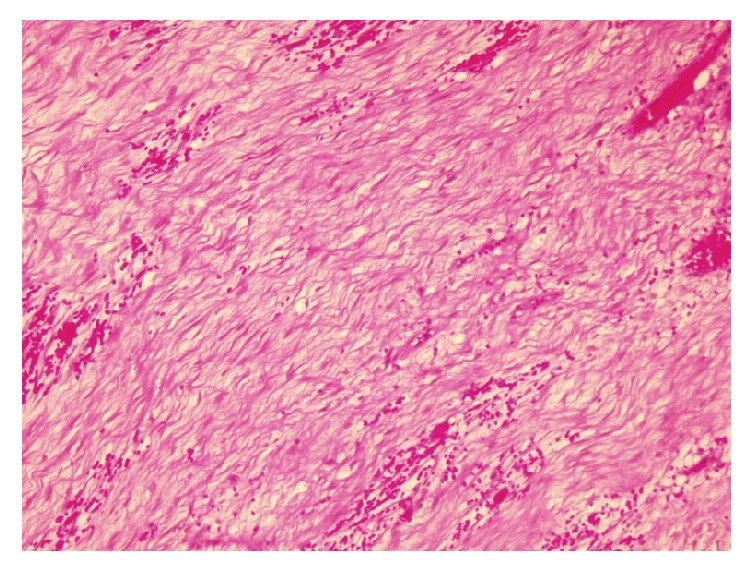
Photomicrograph (H&E stain; original magnification ×10).

**Figure 2:**
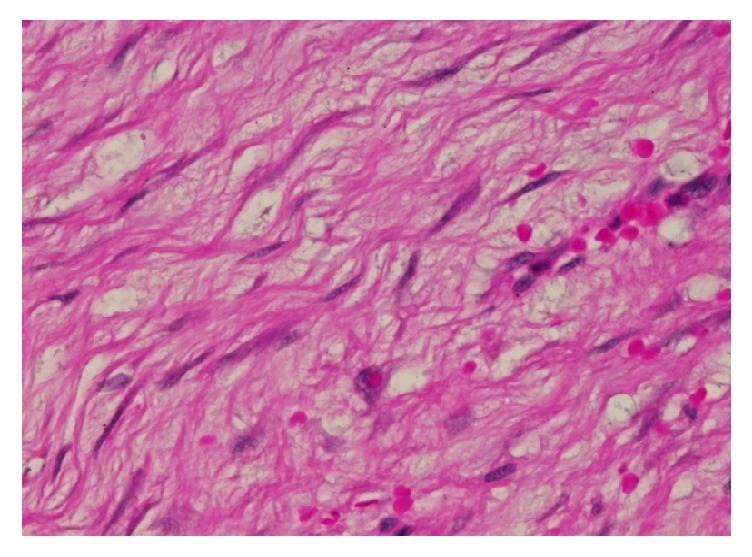
Photomicrograph (H&E stain; original magnification ×40).

**Figure 3:**
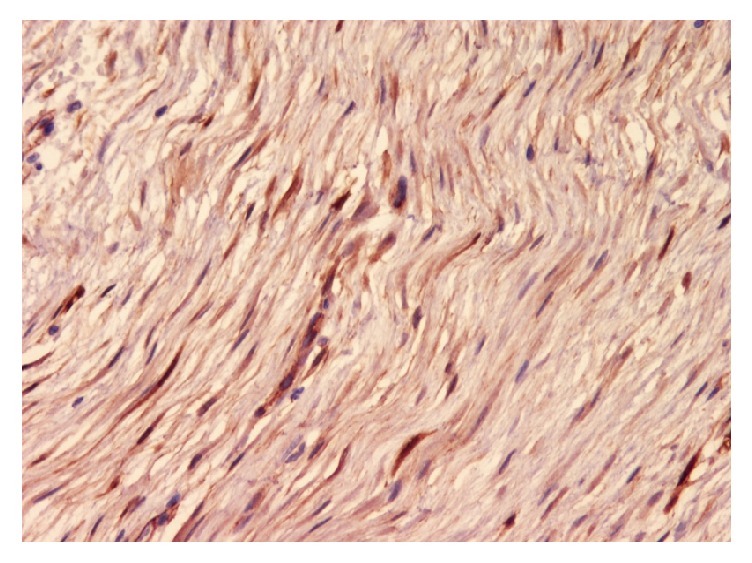
Photomicrograph (IHC stain; original magnification ×40) of tumor cells showing B-catenin positivity.

**Figure 4:**
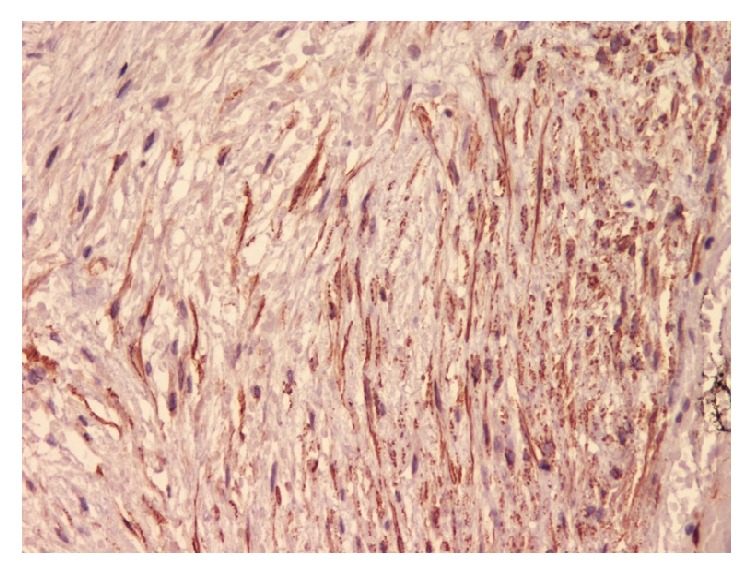
Photomicrograph (IHC stain; original magnification ×40) of tumor cells showing SMA positivity.

**Figure 5:**
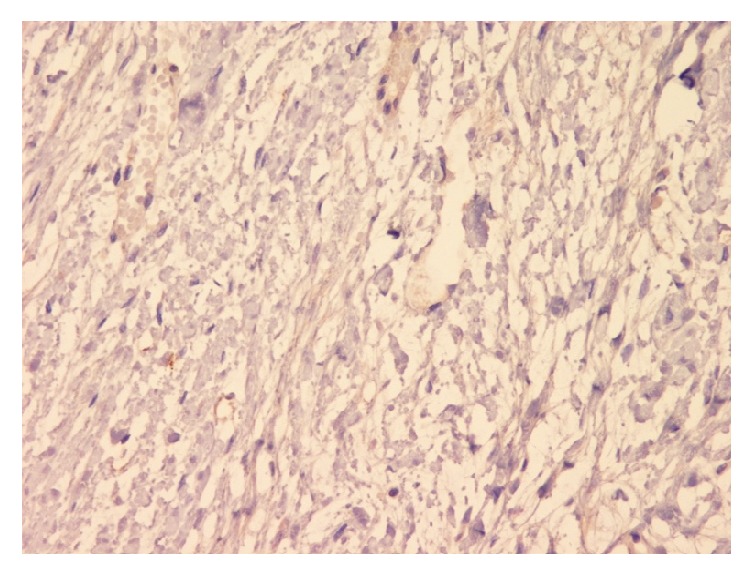
Photomicrograph (IHC stain; original magnification ×40) of tumor cells negative for C-kit (CD117).

**Figure 6:**
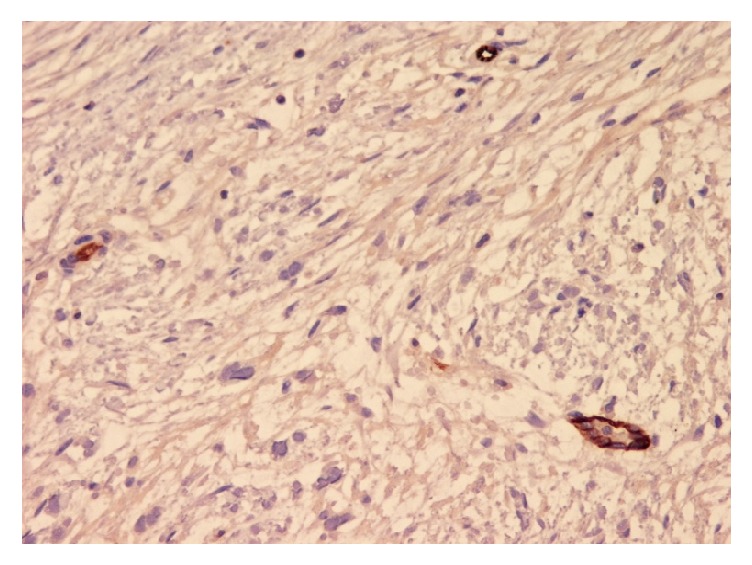
Photomicrograph (IHC stain; original magnification ×40) of tumor cells negative for CD34. Internal control of positivity stained blood vessels is identified.

**Figure 7:**
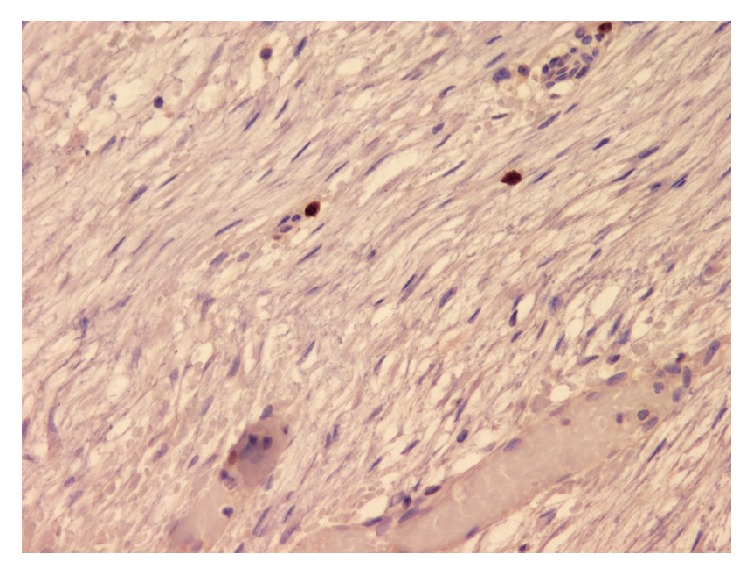
Photomicrograph (IHC stain; original magnification ×40) of tumor cells showing low Ki-67 index.

**Figure 8:**
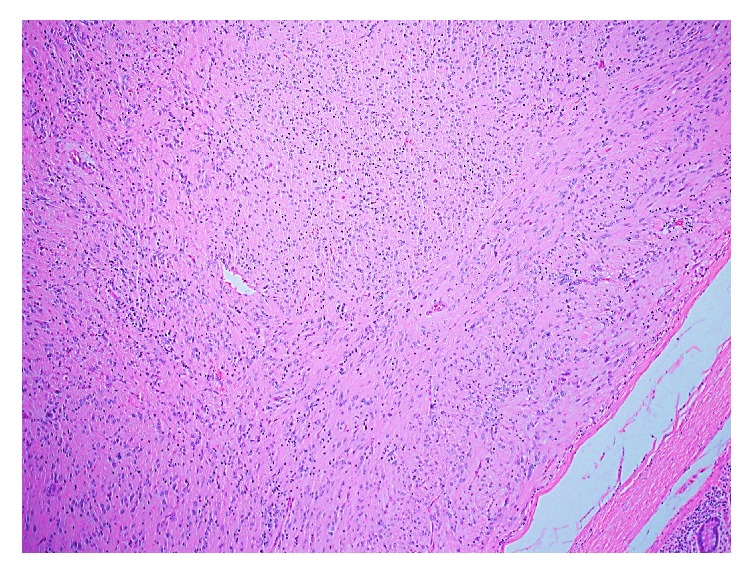
Photomicrograph (H&E stain; original magnification ×10) from previously diagnosed GIST.

**Figure 9:**
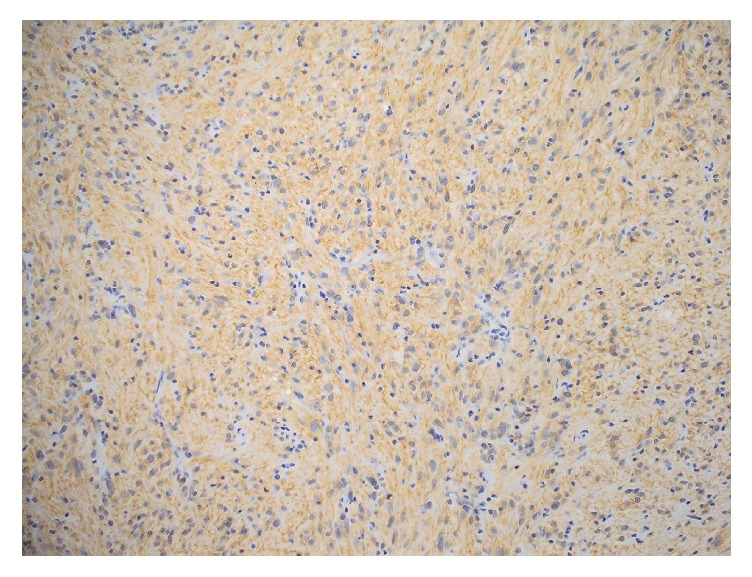
Photomicrograph (IHC stain; original magnification ×20) of tumor cells from previously diagnosed GIST positive for C-kit (CD117).

A 46-year-old Saudi male presented to our hospital in 2016 complaining of vomiting and constipation for two days. The patient had previous history of gastrointestinal stromal tumor (GIST) of the small bowel in 2013. He underwent a small bowel resection in an outside hospital and was given Imatinib therapy until 2015 in the United States of America. The previous H&E (Figure 8) and IHC (Figure 9) slides were reviewed in our hospital for confirmation. Computed tomography (CT) of the abdomen showed an oval-shaped soft tissue density lying anterior to the right external iliac vessels measuring 2.9 × 1.7 cm. It had increased in size when compared to a previous CT. The remaining abdominal organs were unremarkable. No lymphadenopathy was identified. The initial clinical impression was recurrence of GIST. The patient underwent a right hemicolectomy with terminal ileum resection. Macroscopic examination of the specimen revealed a small, firm, well-circumscribed mass in the mesentery of the ilium. It measured 3 × 2 × 1.5 cm. The mass was not grossly invading the ileum. Its cut surface was tan and homogenous with some areas of hemorrhage. It was grossly away from the proximal, distal, and mesenteric margins. The remainder of the specimen was unremarkable. Microscopic sections of the mass revealed a poorly circumscribed growth of spindle cells showing amphophilic cytoplasm and open chromatin with distended nuclear membranes (Figures 1 and 2). The cells were in a collagenous background. No atypia was identified. Rare mitotic figures were seen. The growth is infiltrating the mesenteric fat. Differential diagnoses of Desmoid tumor, GIST, leiomyoma, neurofibroma, and inflammatory myofibroblastic tumor were considered. A panel of the following immunohistochemistry was performed: B-catenin, smooth muscle actin (SMA), C-kit (CD117), CD34, and Ki67. S100, CKPAN, Vimentin, and ALK were considered at first but were not done, as we believed the case was straightforward. By histology, we excluded neurofibroma and inflammatory myofibroblastic tumor, as there was absence of the characteristic wavy nuclei of neurofibroma and the dense inflammation of inflammatory myofibroblastic tumor. Immunohistochemistry showed that the cells were positive for nuclear B-catenin (Figure 3) and smooth muscle actin (SMA) (Figure 4). The cells were negative for C-kit (CD117) (Figure 5) and CD34 (Figure 6). The Ki67 index was less than 5% (Figure 7). The negativity of C-kit (CD117) excluded GIST. The positivity of smooth muscle actin (SMA) included leiomyoma, but it was quickly excluded with the positivity of B-catenin. The final diagnosis of deep fibromatosis (Desmoid tumor) was made. The patient was put on surveillance and had a CT scan done again in April 2017. The CT scan showed no evidence of recurrence or metastasis. Patient is doing well and has no active complaints as of this current time.

## 3. Discussion

Desmoid tumors are a group of proliferative tumors that originate from the musculoaponeurotic stromal elements. They mostly present in adolescence and young adults. The estimated incidence in the general population is two to four per million population per year [1]. Their clinical presentations vary and depend on their location. They may mimic cancer and cause destruction or compression of adjacent organs. Desmoid tumors can arise in any skeletal muscle. They most commonly develop in the anterior abdominal wall and shoulder girdle. They can also develop in the retroperitoneum and mesentery. Risk factors of development include previous history of abdominal surgery, familial adenomatous polyposis (FAP) [2, 3], and Gardner syndrome [4]. Desmoid tumors of the mesentery should raise the suspicion of Gardner syndrome especially after a surgical resection [5]. Estrogen elevation and pregnancy status are also found to be risk factors [6, 7]. On gross examination, these tumors have infiltrative margins and a large, firm, white, and gritty cut surface. Microscopically, they demonstrate poorly circumscribed lesions with infiltrative margins. The neoplastic cells are proliferating fibroblasts and myofibroblasts with reduced amphophilic cytoplasm, open chromatin of the nuclei, well-defined nuclear membrane, and a distinct nucleolus. No atypia is identified. Perivascular lymphocytes at edge of lesion may be seen. Few mitotic figures are identified. The molecular pathogenesis of Desmoid tumors includes mutations in B-catenin or APC [8]. APC is a component of the WNT signaling pathway. A major function of the APC protein is to hold *β*-catenin activity in place. APC forms a destruction complex with B-catenin and prevents its accumulation in the cytoplasm. Signaling by WNT blocks the formation of the destruction complex, allowing *β*-catenin to translocate from the cytoplasm to the nucleus. Once in the nucleus, *β*-catenin forms a transcription activation complex to promote the growth of epithelial cells. Mutations of B-catenin are due to mutations in the encoding gene CTNNB1. A study in 2008 [9] revealed that three discrete mutations in two codons of CTNNB1 exon 3 were identified: 41A, 45F, and 45P. Patients with 45F mutations had higher risk of recurrence. By immunohistochemistry, Desmoid tumors usually have nuclear positivity for B-catenin, which indicates a B-catenin mutation. Although B-catenin has a good reputation of being one of the best stains for Desmoid tumors, one must remember that it is sensitive but not specific for this tumor. They are also positive for vimentin and variably positive for smooth muscle actin and muscle specific actin. CD117 (C-kit) is usually negative, but if positive, Desmoid tumors tend to have a membranous staining pattern [10]. CD34, S100, keratins, and ALK are negative. A study showed that some Desmoid tumors stained positive for PR, ER, and Androgen receptors [11]. This further implicates the theory of a hormonal effect in the pathogenesis. Ultrastructural analysis of Desmoid tumors by electron microscopy shows complete myofibroblastic/fibroblastic differentiation [10]. Prognosis-wise, these tumors are locally infiltrative and aggressive but do not metastasize [12]. They have a high recurrence rate and are mainly treated by surgical resection with wide margins. Some studies reported that Desmoid tumors show a partial response to Imatinib therapy [13, 14]. They can also respond to combined chemotherapy [15].

GIST originates from the Cajal cells, a group of cells which control gut motility. It is the most common mesenchymal tumor of the abdomen. These tumors mostly occur in the stomach, but can occur in the esophagus, colon, rectum, small bowel, omentum, and mesentery. The incidence of GIST is between 11 and 20 per million. The peak age is 60 years but can occur in those of 40 years. Risk factors of developing GIST are related to nonhereditary syndromes like Carney triad, familial GIST syndrome, Carney-Stratakis syndrome, and neurofibromatosis type 1. GIST may be discovered incidentally. It may present as a mass with bowel obstruction, abdominal pain, melena, or blood loss with anemia due to mucosal ulceration. On gross examination, this tumor is large, solitary, and well-circumscribed. The cut surface is whorl-like and fleshy with some cystic, hemorrhagic, or necrotic areas. Microscopically, these tumors can be spindle-celled, epithelioid, or mixed pattern. The spindle-celled form shows proliferation of bland spindle cells with pale to eosinophilic fibrillar cytoplasm growing in short fascicles. Nuclear palisading may be identified. Perinuclear vacuoles may be seen. The stroma is extensively hyalinized. Minimal pleomorphism and a mitotic rate of <2 mitotic figures/50 HPFs are identified. In GIST epithelioid form, sheets of epithelioid cells with round small nucleoli are seen. The cytoplasm is eosinophilic and dense. The stroma is hyalinized. Only rare mitotic figures are seen. The mixed pattern has a mixture of both patterns. The molecular pathogenesis of these tumors includes gain-of-function mutations in C-kit and PDGFRA, which are both tyrosine kinase receptors. They are mutually exclusive. The activation of the tyrosine kinase receptor produces intracellular signals which promotes tumor cell proliferation. Mutations of C-kit are found in most cases of GIST. A subset of GIST carries BRAF mutations [19] and loss-of-function mutations of succinate dehydrogenase complex [20] as well. By immunohistochemistry, a confirmatory cytoplasmic staining for C-kit (CD117) [10] is recommended to make the diagnosis of GIST. These tumors are also positive for vimentin, CD34, and DOG1 and variably positive for smooth muscle actin. Desmin and S100 are negative. Ultrastructural analysis by electron microscope of GISTs reveals incomplete smooth muscle differentiation [10]. Prognosis of GISTs depends on their size, mitotic rate, and location. GISTs of the small intestine are the most aggressive while gastric GISTs are the least aggressive. These tumors are considered malignant and can metastasize distally. GIST has also been known to synchronously occur with other neoplasms, with adenocarcinoma being the most common as reported in a case series [21]. The mainstay of treatment for GIST is surgical resection. Postsurgical treatment with a tyrosine kinase inhibitor Imatinib (Gleevec) is reserved for those with positive C-kit and PDGFRA mutations.

Most case reports present cases of diagnosed GIST with subsequent development of Desmoid tumor. Most Desmoid tumors develop in the mesentery or near the area of the previously resected GIST. In rare cases, the Desmoid tumor may develop in an unrelated area. The relationship between Desmoid tumors and GIST has been studied and documented in literature. A cohort study of 28 cases suggests that there is a nonrandom association between Desmoid tumors and GIST [22]. The reason why both these entities are associated with each other is currently unknown. We suggest that the association has to do with their cell origin. The origin of Desmoid tumors is myofibroblasts. These cells have a phenotype between fibroblasts and smooth muscle cells. Although their main function is fibrosis, they also have the ability to contract during wound healing. The origin of GIST is the Cajal cell which is found in many types of smooth muscle tissue. They function as a pacemaker to create slow contractions of smooth muscle in the GI tract. In conclusion, one could say that these two cells are related and perhaps this is why these tumors are associated with each other. The time interval between the development of these two tumors varies greatly from months to years after resection. A study showed an average time of 30 months [22] between these two tumors.

In our presented case, this patient had developed a Desmoid tumor in a previous location of a resected small bowel GIST after 3 years. When compared with other case reports, our case falls in the average category (Table 1). It is the first case of GIST with subsequent development of Desmoid tumor to be reported in Saudi Arabia.

In conclusion, Desmoid tumors (deep fibromatosis) and GISTs are both different and rare neoplasms. It is critical to differentiate Desmoid tumors from GIST due to their different biological behavior and treatment methods. Although literature has observed an association between these two entities [16, 18, 23], they have different pathogenesis. Desmoid tumors can be easily mistaken for GIST as both tumors overlap in clinical presentation, morphology, and immunohistochemistry. We recommend further studies to pinpoint why both tumors are associated with each other.

## Figures and Tables

**Table 1:** Presented case (1) compared to other case reports.

Case number	Sex	Age	Location of GIST	Location of subsequent Desmoid tumor	Time interval between the two entities (months)	Management
(1)	Male	46	Small bowel	Small bowel	36	Resection and Imatinib
(2) [16]	Male	54	Jejunum	Retroperitoneum	36	Resection only
(3) [16]	Male	45	Duodenum	Duodenum	12	Resection and Imatinib
(4) [17]	Male	62	Antrum	Infrapyloric, mesentery	48	Resection and Imatinib
(5) [18]	Male	37	Gastric	Mesentery	11	Resection and Imatinib
